# Emotional Autobiographical Memory Associated with Insular Resection in Epileptic Patients: A Comparison with Temporal Lobe Resection

**DOI:** 10.3390/brainsci11101316

**Published:** 2021-10-03

**Authors:** Mélanie Descamps, Olivier Boucher, Dang Khoa Nguyen, Isabelle Rouleau

**Affiliations:** 1 Département de psychologie, Université du Québec à Montréal, Montreal, QC H2X 3P2, Canada; descamps.melanie@gmail.com; 2 Centre de recherche du Centre Hospitalier de l’Université de Montréal, Montreal, QC H2X 0A9, Canada; olivier.boucher@umontreal.ca (O.B.); d.nguyen@umontreal.ca (D.K.N.); 3 Service de psychologie, Centre Hospitalier de l’Université de Montréal, Montreal, QC H2X 0C1, Canada; 4 Département de psychologie, Université de Montréal, Montreal, QC H3C 3J7, Canada; 5 Service de neurologie, Centre Hospitalier de l’Université de Montréal, Montreal, QC H2X 3E4, Canada

**Keywords:** autobiographical memory, emotion, insula, temporal lobe, epilepsy, neurosurgery

## Abstract

The insula is involved in a wide variety of functions, including social and emotional processing. Despite the numerous connections it shares with brain structures known to play a role in autobiographical memory (AM), little is known on the contribution of the insula to AM processing. The aim of the study was to examine emotional AM retrieval in patients with insular resection for drug-resistant epilepsy. Ten patients who underwent partial or complete insular resection (IR) were matched on age, sex, and education, to fifteen patients who underwent temporal lobectomy (TL), and to fifteen healthy controls. Participants were asked to recall four positive, four negative, and four neutral memories from their past using the autobiographical interview procedure. The results suggest that AM for emotional and neutral events after IR was comparable to that of healthy controls, whereas deficits were observed after TL. However, an independent examiner judged IR patients’ memories as poorer than those of healthy controls on the episodic richness scale, suggesting a lack of some aspects of rich and vivid remembering. Furthermore, analysis on subjective self-rated scales revealed that, contrary to healthy controls, patients with IR judged their neutral memories as more emotional. This study suggests that AM is generally preserved after IR. However, given the small sample size and varied lesion location, one cannot totally exclude a potential role of specific insular sub-regions on some aspects of autobiographical memory. In addition, IR patients showed poor emotional judgment for neutral memories, which is congruent with previous findings of altered emotional processing in this population.

## 1. Introduction

The insula is often considered as the fifth lobe of the brain. Located at a crossroad between the temporal, frontal and parietal opercula, it has multiple connections to other areas of the brain [1]. Although its involvement in vicero-sensory and vicero-motor processing has been established for decades [2], its role in cognitive functioning has long been neglected. However, growing interest in the past decade [3] has shown that the insula is involved in a wide variety of functions, including auditory processing, vestibular function, pain and temperature perception, speech initiation and speech production, body representation, time perception as well as social emotional processing [4,5,6].

In recent years, insular function has consistently been linked with processing, feeling and the recognition of emotions [7,8,9,10]. For example, Boucher et al. [11] demonstrated an impairment in facial recognition of happiness, fear and surprise after insular resection for the control of epilepsy. In a case study, Borg et al. [12] found reduced emotional intensity perception in facial emotion recognition task in a patient with a lesion to the left posterior insula, whereas recognition of all emotions, except for disgust, was preserved. Berntson et al. [13] assessed patients on an emotional task in which patients with an insular lesion and patients with an amygdalar lesion had to rate valence (very positive, moderately positive, neutral, moderately negative and very negative) and affective arousal on picture stimuli. They found reduced arousal and an attenuation of valence rating in patients with insular lesions to both negative and positive stimuli, whereas patients with amygdalar lesions only showed attenuated arousal for negative stimuli and preserved valence ratings compared to controls. The authors concluded that the insula may be involved in recognition, processing, the assignment of valence and affective arousal, whereas the amygdala might have a more restricted role in affective arousal, especially for negative stimuli. The number of studies on the role of the insula in emotional processing is increasing; however, its role in other functions involving emotions, such as emotional memory, remains unclear.

Animal studies have shown the insula to be involved in social recognition memory [14], taste memory [15], recognition memory formation and consolidation [16,17]. In humans, neuropsychological assessment after insular damage has shown inconsistent results regarding episodic memory [18,19]. In a case study using fMRI, Borg et al. [20] found the insula to be activated in the recall of painful personal events versus non-painful personal events in a patient with a history of chronic pain. In a PET study with healthy participants, Fink et al. [21] found the right insula to be involved in the autobiographical memory network. To date, the insula remains poorly explored in autobiographical memory studies [22]. To the best of our knowledge, no study has yet explored emotional autobiographical memory in patients with insular damage. However, given the numerous connections between the insula and structures known to play a role in the autobiographical memory network, such as the prefrontal and orbitofrontal cortices, the entorhinal cortex, the parahippocampal complex, the hippocampus and the amygdala [23,24,25,26], it is plausible that the insula is involved in this domain of mnesic functions, especially for emotional autobiographical memories (AMs).

By contrast, autobiographical memory has been extensively studied in patients with temporal lobe epilepsy and after temporal lobe epilepsy surgery, because the hippocampus is a core structure of the AM network. In this population, personal episodic memory is known to be impaired, whereas personal semantic memory usually appears to be preserved [27,28,29,30,31,32,33,34]. Personal episodic information is usually described as a unique event that is specific in time and place, entails sensory information and evokes a vivid remembering of the event, whereas personal semantic memory refers to repeated events and general facts about oneself. Many AM studies entailed various time periods, usually covering the entire life-span, although recent memories and memories of the ‘first time period’ are known to be better recalled [35].

In recent years, insulo-opercular epilepsy has been recognized as a distinct form of epilepsy, and the number of insular cortectomies as treatment for drug-resistant insular epilepsy has been increasing [36,37,38]. However, whether resection of the insular cortex is associated with disturbance in emotional AM remains unknown. The aim of this study was to examine emotional AM retrieval in patients with insular resection (IR) for drug-resistant epilepsy, and at the same time, further our knowledge of the insula. Personal episodic and semantic components of autobiographical memory were explored for emotional and neutral personal events. Patients with IR were compared to two control groups; a group of healthy participants and a lesion-control group of patients who underwent temporal lobectomy, sparing the insula, for the control of drug-resistant epilepsy. Well-known deficits in patients after temporal lobe resections as well as facilitated access to patients who underwent such surgical procedures (because they are relatively common) offer a valuable opportunity to examine and compare the effects of insular resections. We expect poorer performance after IR compared to healthy controls on emotional memories but better performance after IR than after TL. Neutral memories should remain preserved after IR.

## 2. Materials and Methods

### 2.1. Participants and Procedure

A total of 10 patients who underwent unilateral (5 left and 5 right hemisphere) resection of the insular cortex for drug-resistant epilepsy were matched with two control groups on age, sex and education: 15 healthy control participants and a lesion-control group of 15 patients who underwent temporal lobectomy (TL) sparing the insula (7 right and 8 left). Beyond offering a basis for comparison with a common type of epilepsy surgery known to affect AM, inclusion of this group ensures that our AM assessment method is valid and sensitive to AM deficits. All participants were aged between 23 and 50 years. Patients were recruited at the Centre hospitalier de l’Université de Montréal (CHUM). All patients except one were tested at least 18 months after surgery. Surgical information is provided in Table 1.

In order to control for potential confounding factors, a brief neuropsychological assessment was performed, including measures of verbal (Similarities) and visual reasoning (Matrix Reasoning) from the WAIS-IV [40], processing speed (Coding) from the WAIS-IV, an alternative version of Logical Memory [41], a simplified computerized facial emotion recognition test [11], and the BDI-II questionnaire to assess depressive symptomatology (Beck’s Depression Inventory).

The CHUM institutional ethics committee approved this study. Informed written consent was obtained from all participants. Each participant received CAD 50 in financial compensation at the end of the assessment.

### 2.2. Autobiographical Interview

Autobiographical memory was assessed using an adapted version of the Autobiographical Interview described by Levin et al. [42]. Instructions, scales and event list were translated in French using back translation. The interview was adapted for time period and valence. We replaced the original five time periods with two: from 16 to 25 y/o (remote) and from the last year except the past two weeks (recent). The remote period was designed to target events that happened before surgery and the usually observed ‘bump’ of memories covering the ‘first time period’ [35], whereas the recent period targeted memories from events that occurred after the surgery. The remote period was adjusted when the patient had surgery before the age of 25 years (memories between 16 y/o and surgery) and when the participant was aged under 25 years (memories between 16 y/o and current age minus 18 months). For each time period (remote and recent), participants were asked to retrieve 2 memories per valence (2 positives, 2 negatives and 2 neutrals), resulting in a total of 12 memories. Two memories were requested per condition to avoid targeting only the most accessible event of a condition (as discussed in [43,44]). Neutral memories were defined to participants as being unimportant, consequence-free and emotion-free events, such as bringing the dog to the groomer, making a purchase, visiting a museum, etc.

Participants were instructed to freely recall memories from events of their choice in which they were personally involved; events had to be unique, specific to a time and place, and had to have occurred over a short time period of less than one day. When participants struggled with finding an event to recall, a list of typical life events was provided. The autobiographical interview entails three recall conditions. During free recall, participants were allowed to speak without interruption until the narration reached a natural end. The interviewer then administered general probing, when necessary, in order to encourage participants to develop the story or clarify instructions. Finally, a specific recall condition, consisting of a semi-structured interview designed to elicit episodic details from different aspects of memories (time, place, odors, visual images, thoughts, etc.), was administered. The order of condition administration was randomized per the valence and age of memories. Narratives were audio-taped, transcribed into a text document and made anonymous.

The scoring procedure is described in Levine et al. [42] and is summarized in Table 2. Narratives were segmented into pieces of information (details). First, details were classified as either internal or external. Internal details describe the main unique event as defined above and are believed to represent the episodic component of AM. External details entail all information that does not directly pertain to the main event, such as general knowledge, factual facts, part of another event or repetitions, and relate to the semantic component of AM. Internal and external details were then divided into more specific categories (event, time, place, perceptual, emotion/thought); semantic details or repetitions counted as external details. Final scores are expressed in terms of the number of details recalled, either as internal vs. external and for each specific category. In order to control for speech fluency, we computed an internal-to-total details ratio. Scores were computed after the specific probe (free recall + general probe + specific probe). The scorer (M.D.) was trained using the training program kindly supplied by Dr. Brian Levine with the AI protocol. The trained scorer’s classification was compared with those of experimented scorers on a set of memories to assess inter-rater reliability; reliability coefficients were all above 0.90. Data entry was carried out with a Python3 script designed for this study.

### 2.3. Ratings by an Independent Scorer

As per the AI protocol, each memory was rated by an independent rater (E.H.), blind to the participant’s group on time integration (3-point scale), episodic richness (6-point scale) and the autobiographical memory interview (AMI; 3-point scale) [45]. The scoring procedure is described in Table 3. Only ratings after the specific probe are reported. Examiner rating was considered only for recalled memories.

### 2.4. Subjective Self-Rated Scales

After each recall, participants were asked to rate on a −6 (extremely negative) to 6 (extremely positive) point scale how positive or negative the event was when it occurred, with 0 corresponding to neither pleasant nor unpleasant. All participants judged negative memories as negative and positive ones as positive; therefore, all ratings from negative memories were multiplied by −1, resulting in a 0–6-point scale (emotional intensity scale). As per the AI protocol, participants were asked to rate their memories on 1–6-point scales in terms of how clearly they could visualize the event (visualization scale), how much their emotional state changed from before to after the event occurred (emotional change scale), how personally important this event was for them when it happened (importance at the time scale), how personally important this event is now (importance today scale) and how often they think or talk about this event (rehearsal scale).

### 2.5. Statistical Analysis

Between-group comparisons were performed using one-way analyses of variance (ANOVAs) on demographics and control variables. Between-group differences in sex were assessed using a Chi2 test. Mixed factorial ANCOVAs with a linear mixed model approach with BDI raw score as a covariate were used to compare groups and valence on the experimental task. Using a mixed model approach in factorial mixed ANOVA allows for greater power due to the repeated measures in each group condition (IR *n* = 40; TL minimum *n* = 54; Healthy participant *n* = 60). Post hoc comparisons were performed using the Bonferroni correction test. We inspected the residual distribution for normality and outliers; skewness and kurtosis were checked using the normality range −1.5 to 1.5. Five variables were transformed using logarithmic transformation; total internal and external details, and event, perceptual, emotion/thought details; following logarithmic transformations, residues for all five dependent variables were normally distributed. Missing data were handled through maximum likelihood, because this procedure utilizes all existing data and has been shown to be superior to listwise and pairwise deletion [46]. Each participant had at least one memory in each category, and all participants were included in the analyses, ensuring that missing data did not introduce any bias.

Statistical analyses were carried out using SPSS Statistics software version 25.0 (SPSS, Chicago, IL, USA). Differences were considered significant at *p* < 0.05.

## 3. Results

### 3.1. Study Sample

The three groups did not differ significantly on mean age, education, IQ-associated measures (verbal and visual reasoning), processing speed, emotion recognition or epilepsy-related factors in patients (Table 4). Groups differed on the episodic verbal memory task after immediate (F[2,37] = 8.64, *p* = 0.001, η^2^ = 0.318) and delayed (F[2,37] = 17.29, *p* < 0.001, η^2^ = 0.483) recall. Post hoc comparisons revealed that both the patients with IR and patients with TL showed poorer performance than healthy controls on immediate (*p* = 0.016, and *p* = 0.001, respectively) and delayed recall (*p* = 0.016, *p* < 0.001, respectively). No significant differences were found between the two patient groups on immediate (*p* = 0.810) and delayed (*p* = 0.063) recall, although patients with IR tended to perform better on the latter. Comparisons between groups on the BDI-II questionnaire revealed a significant difference (F[2,37] = 4.55, *p* = 0.017, η^2^ = 0.197): post hoc comparison revealed that patients with TL had significantly more depressive symptoms than healthy controls (*p* = 0.013), whereas patients with IR did not differ from either healthy controls (*p* = 0.472) or TL patients (*p* = 0.296). Depression is known to impact memory performance [47]; therefore, AI score analyses were conducted with BDI-II scores as a covariate.

### 3.2. Age of Memories—Time Periods

Mixed factorial ANCOVAs on the number of *internal details, external details and internal-to-total ratio* revealed no influence of time period (remote vs. recent). No significant interaction was found between group, valence and time period (F[4,429] = 1.93, *p* < 0.104; F[4,429] = 0.54, *p* < 0.710; F[4,428] = 0.97, *p* < 0.426, respectively) or group and time period (F[2,429] = 0.76, *p* < 0.468; F[2,429] = 2.46, *p* < 0.087; F[2,428] = 1.09, *p* < 0.336, respectively) in any recall condition. Time periods did not explain any of the group differences; therefore, the time period factor was dropped from further analyses.

### 3.3. Number of Memories Retrieved

All patients with IR and healthy participants were able to recall the twelve memories asked, whereas only 11 out of 15 patients with TL were able to fully complete the task. In the TL group, difficulties appeared for negative and neutral events, but not for positive events. Thus, in the TL group, further analyses were conducted using recalled events (positive, *n* = 60; negative, *n* = 54; neutral, *n* = 55). Each participant had at least one memory in each valence category.

Unexpectedly, neutral memories appeared qualitatively more emotionally charged in patients with TL than in patient with IR and healthy participants. Examples of neutral memories recalled can be found in Table 5.

### 3.4. Autobiographical Memory Performance

Table 6 summarizes group performance on the number of details recalled (internal, external, internal-to-total ratio, each specific detail category) and ratings by an independent examiner (time integration, episodic richness and AMI scales).

#### 3.4.1. Internal Details

Mixed factorial ANCOVAs on the number of *internal details* revealed a significant two-way interaction between groups and emotional valence (F[4,429] = 5.22, *p* < 0.001, η^2^ = 0.106). Negative memories (F[2,64] = 16.45, *p* < 0.001) were more richly recalled by healthy controls than by both patient groups, but patients with IR still performed significantly better than patients with TL. For positive (F[2,63] = 8.97, *p* < 0.001) and neutral (F[2,64] = 4.84, *p* = 0.011) memories, IR patients and healthy controls retrieved significantly more internal details than TL patients, and no difference was found between IR patients and healthy controls.

#### 3.4.2. Internal-to-Total Ratio

Using the *internal-to-total ratio*, mixed factorial ANCOVAs revealed a significant two-way interaction between groups and emotional valence (F[4,429] = 2.96, *p* = 0.020, η^2^ = 0.067). Negative memories (F[2,77] = 12.76, *p* < 0.001) were still better recalled by patients with IR and healthy participants than by patients with TL. Using the internal-to-total details ratio, the difference between IR patients and healthy participants was no longer significant for negative memories. However, for positive memories (F[2,75] = 6.47, *p* = 0.003), healthy participants still recalled personal events more richly than TL patients, but IR patients tended to range between the two other groups; thus, we failed to find a statistical difference between patients with IR and healthy controls or patients with TL. Differences between groups did not reach significance for neutral memories (F[2,77] = 2.90, *p* = 0.061).

#### 3.4.3. External Details

No significant interaction was found between groups and emotional valence on external details (F[4,429] = 0.58, *p* = 0.678, η^2^ = 0.023). No group main effect was found; all three groups recalled an equivalent number of external details (F[2,40] = 0.55, *p* = 0.581, η^2^ = 0.027).

#### 3.4.4. Types of Details

Group differences were examined on the number of details for each internal detail category (Event Details, Time, Place, Perceptual and Emotion/Thoughts) using mixed factorial ANCOVAs. Analyses revealed a significant Group–Valence interaction for event details (F[4,429] = 3.65, *p* = 0.006, η^2^ = 0.094) and emotion/thought details (F[4,429] = 4.37, *p* = 0.002, η^2^ = 0.103). For positive (F[2,71] = 9.78, *p* < 0.001), negative (F[2,73] = 14.27, *p* < 0.001) and neutral (F[2,72] = 4.66, *p* = 0.012) memories, event details were significantly better recalled in the healthy control group and IR group compared to TL patients, and no difference was found between IR patients and healthy controls. Emotion/thought details were more richly recalled in the control group compared to TL patients for positive (F[2,67] = 3.75, *p* = 0.029) and negative memories (F[2,68] = 7.93, *p* = 0.001), but not for neutral memories (F[2,68] = 2.35, *p* = 0.104), and patients with IR tended to fall between the two other groups with no significant differences. Significant group main effects were found for time (F[2,40] = 11.55, *p* < 0.001, η^2^ = 0.368) and perceptual details (F[2,40] = 5.51, *p* = 0.008, η^2^ = 0.218). Controls and IR patients recalled significantly more time details than TL patients. Perceptual details were significantly better recalled in the control group compared to TL patients. However, no significant difference was found between patients with IR and the two other groups. No main effect of group was found for place details (F[2,40] = 0.85, *p* = 0.437, η^2^ = 0.035).

### 3.5. Ratings by an Independent Examiner

Scales rated by an independent examiner on time integration, episodic richness, and AMI rating were analyzed using mixed factorial ANCOVAs. Time integration (F[4,430] = 1.53, *p* = 0.194, η^2^ = 0.045) and AMI ratings (F[4,428] = 0.70, *p* = 0.595, η^2^ = 0.017) revealed no significant group by valence interaction. However, main effects of group were significant for time integration (F[2,40] = 13.45, *p* < 0.001, η^2^ = 0.390) and AMI rating (F[2,38] = 12.07, *p* < 0.001, η^2^ = 0.353). IR patients were not different from healthy controls on either scale and the examiner found recall among patients with IR and healthy participants to be more integrated in time and better remembered on AMI criteria than that of TL patients.

When looking at the episodic richness scale, analyses revealed a significant group–valence interaction (F[4,430] = 3.13, *p* = 0.015, η^2^ = 0.104). For negative (F[2,85] = 18.20, *p* < 0.001) and positive (F[2,82] = 10.97, *p* < 0.001) memories, IR and TL patients’ memories were judged to be poorer in episodic details compared to healthy controls. Differences between IR and TL patients were not significant. For neutral memories (F[2,85] = 3.72, *p* = 0.028), memories of TL patients were found to be poorer than those of healthy participants, but patients with IR did not differ from the two other groups.

### 3.6. Subjective Self-Rated Scales

Results on subjective self-rated scales (emotional intensity, visualization, emotional change, importance at the time, importance today and rehearsal scale) can be found in Table 7. Mixed factorial ANCOVAs on self-rated scales revealed significant group–valence interactions for emotional intensity (F[4,430] = 7.09, *p* < 0.001, η^2^ = 0.137), importance at the time of the event (F[4,429] = 10.84, *p* < 0.001, η^2^ = 0.193) and emotional change (F[4,430] = 5.18, *p* < 0.001, η^2^ = 0.128). No difference was found for positive and negative events on any of these three scales. However, patients with IR and patients with TL rated their memory for neutral events as significantly more emotionally intense, more important at the time, and inducing greater emotional change state at the time of the event than controls. No main effect of the group was found on importance today (F[2,40] = 0.45, *p* = 0.642, η^2^ = 0.024), visualization (F[2,40] = 0.29, *p* = 0.749, η^2^ = 0.016) or rehearsal (F[2,40] = 0.44, *p* = 0.645, η^2^ = 0.020)

## 4. Discussion

The aim of the study was to examine emotional AM retrieval in patients with insular resection for drug-resistant epilepsy. More specifically, it explored, for the first time, the quality of emotional autobiographical memories associated with insular resection compared to healthy controls and a lesion-control group of patients who had temporal lobe epilepsy surgery known to show impairment in remembering personal past episodes.

First, patients with IR had no difficulties completing the task and recalling the twelve memories requested. In comparison, patients with TL showed difficulties with retrieving memories, especially for negative and neutral events, even when they were provided a list of typical events to assist them. Overall, when controlling for speech fluency using the internal-to-total ratio, autobiographical memory performance was preserved for personal episodic and personal semantic memory in IR patients. They did not show significant difficulties retrieving highly detailed memories even though the amount of detail remained slightly reduced compared to healthy controls. By contrast, TL patients showed impairments in retrieving episodic personal details compared to healthy controls and IR patients, but similarly preserved personal semantic details. This pattern of results is consistent with previous findings in temporal lobe epilepsy or after temporal lobectomy; indeed, several studies have shown that TL patients experience difficulties recalling rich and vivid detailed autobiographical memories [27,28,29,30,31,32,33,34]. This is not surprising, because the hippocampus is believed to support the encoding and retrieval of richly detailed episodic information of past personal events [48,49,50]; see Alvarez and Squire [51] for an alternative model of autobiographical memory functioning. In regard to the specific emotional valence of memories recalled, patients with TL were impaired in retrieving episodic details compared to healthy controls for all emotional memories, whereas patients with IR only differed from healthy controls for negative events. However, when controlling for speech fluency using the internal-to-total details ratio, this difference was no longer significant. Results suggest that insular resection does not affect overall episodic autobiographical memory for emotional and neutral events. However, given the small sample size and varied lesion location, one cannot totally exclude a potential role of specific insular sub-regions on some aspects of autobiographical memory.

When looking at examiners’ ratings, memories of healthy participants and IR patients were rated as richer on the time integration scale and on the AMI scale. This is congruent with results on quantitative episodic details. However, memories of healthy controls were rated as richer on the episodic richness scale compared to those of IR and TL patients. The latter results suggest that although patients with IR recalled enough episodic/internal details to reach normality range, they lacked some aspects of rich and vivid remembering to evoke an impression of truly re-experiencing. In order to elucidate why IR patients’ memories were both preserved in terms of episodic details, although also judged as poorer than those of the healthy controls on the episodic richness scale, we then explored each internal detail category separately (event, time, place, perceptual and emotion/thought details). Patients with IR and healthy controls recalled significantly more event and time details than patients with TL; however, IR patients tended to range between healthy controls and TL patients for perceptual and emotion/thought details. Here, although IR patients’ performance did not reach the deficit level when compared to controls, they still recalled slightly fewer perceptual and emotion/thought details than expected. Sensory-perceptual details are a core feature of a vivid recollection of an autobiographical event [52,53,54]; therefore, it is not surprising that even a slight lack of perceptual elements in IR patients’ memories could have impoverished the impression of true re-experiencing or remembering in the independent rater’s perspective on the episodic richness scale. Interestingly, experiments on the remember/know paradigm in laboratory have shown the insula to be involved in the true remembering experience [55], suggesting it is involved in episodic retrieval processing in some way. However, the specific role of the insula in this process remains unclear. Our results suggest that the insula is not a key structure in autobiographical memory, although it could be involved in retrieving emotional and perceptual information.

Finally, contrary to previous studies showing an attenuation of arousal ratings in an emotion recognition task [12] and a visual emotion task [13] in patients with insular lesions, we found that patients with IR judged their neutral memories as more emotionally charged than healthy controls on three out of the six self-reported scales. This was also observed in patients with TL: they both judged their neutral memories as more emotionally intense, more important at the time of the event and source of emotional change at the time of the event than healthy controls. However, qualitatively, when looking at neutral memories that were judged emotionally intense, it seems that TL patients recalled emotional memories instead of neutral ones (buy a new car, get hired, move alone for the first time, car accident, severe incident with a rented car, get kicked out of the family home) whereas patients with IR seemed to judge neutral memories as more emotionally intense than expected for a priori neutral events (buy a TV, take a driving lesson, take a pet to the vet for check up, go to a restaurant, help someone find a new car, play in a jam session, get a ticket, go to water slides). Here, it is possible that, on the one hand, TL patients’ judgment was accurate but they recalled emotional memories instead of neutral ones, probably because emotional memories are more accessible. On the other hand, patients with IR did recall more neutral memories but assigned stronger emotional intensity to them. However, formal evaluation would be necessary to confirm our findings. Future studies could explore this hypothesis using script analysis techniques such as *Linguistic Inquiry and Word Count* [56], already tested in emotional autobiographical memory research [57,58]. This technique allows for a count of emotional words used in narratives and could help clarify if participants recalled emotional memories instead of neutral ones. We found no difference between groups in emotional judgment of emotional memories, suggesting that cognitive evaluation of valence is preserved and patients recalled emotional events congruent with instructions. Difficulties seem to appear when the emotional context is ambiguous. Our results support the idea that the insula is involved in the recognition and processing of affective arousal [13].

Some limitations must be taken into account when considering the results. Patients were not assessed before surgery; therefore, we cannot evaluate changes specifically induced by surgery. Additionally, functional reorganization may have introduced a bias, because time since surgery varied from one to ten years in our sample; however, patient groups did not differ on this variable. In addition, compensatory neuroplasticity may differ depending on the characteristics of the brain tissue resected (volume, location, neuropathology, etc.). Another limitation, common to all studies of AM, is the subjective nature of autobiographical memories, emotions and valence. We acknowledge that our sample size is small and the precise location and extent of the insular resection varied among patients. Functional segregation occurs in the insula [59]; therefore, we cannot exclude the possibility that resection of specific portions of the insula (e.g., the anterior–ventral part) leads to impairments in emotional AM; however, our sample was too small to compare performance according to the specific insular region resected. Nonetheless, significant differences observed between IR and TL patients suggest that the insula is not as involved as temporal lobe structures in emotional autobiographical memory. Thus, we are confident that our findings represent a valuable contribution to our understanding of emotional AM after insular lobe resection.

## 5. Conclusions

We studied, for the first time, emotional autobiographical memory in patients who underwent insular resection for the treatment of drug-resistant epilepsy. Our results revealed preserved autobiographical memory for emotional and neutral events, which is congruent with the absence of subjective complaints in clinical settings after insular surgery. However, as previously suggested in studies evaluating various emotional judgment tasks, emotional judgment appeared to be impaired for neutral memories.

## Figures and Tables

**Table 1:** Description of IR and TL patients’ characteristics.

Patient	Age at First Seizure (Years)	Age at Surgery (Years)	Time Since Surgery (Years)	Pre-Surgery MRI	Resection Side	Insular Location and Other Areas	Classification of Outcome
a							
IR1.	22	36	6	Normal	R	Posterior + Op TP	Class IV
IR2.	14	32	5.8	CD Op P	R	Posterior + Op P	Class I
IR3.	5	23	6.2	Insular Tuber	R	Complete + Op TPF	Class III
IR4.	5	38	6.2	Normal	L	2/3 Anterior + Op FT	Class I
IR5.	4	33	4.7	Insular CD	L	Superior Posterior + Op P	Class I
IR6.	27	37	7.1	Normal	R	2/3 Anterior + Op F	Class I
IR7.	2	49	1	Insular CD	L	Complete	Class I
IR8.	16	32	2.3	Normal	L	2/3 Anterior + OFC	Class I
IR9.	9	27	7.3	Normal	R	Anterior Inferior + OFC	Class I
IR10.	33	40	9.7	Normal	L	2/3 Anterior + Op T	Class III
b							
TL1.	33	44	1.5	Normal	R	ATL	Class I
TL2.	25	29	3.1	R HS	R	SAH	Class I
TL3.	28	30	4.4	Normal	R	ATL	Class I
TL4.	25	29	5.4	Normal	R	ATL	Class III
TL5.	17	19	7.9	R HH	R	ATL	Class I
TL6.	2	43	6.1	R HS	R	SAH	Class I
TL7.	17	24	3.8	R CD	R	ATL	Class I
TL8.	3	26	3.6	L HS	L	ATL	Class III
TL9.	33	37	4	B HS	L	ATL	Class I
TL10.	11	33	2	L HI	L	ATL	Class I
TL11.	17	34	8.9	Normal	L	SAH	Class I
TL12.	1	26	3.4	L HS	L	ATL	Class III
TL13.	22	24	5	L HA	L	ATL	Class I
TL14.	13	39	0.6	L HS	L	ATL	Class I
TL15.	1	19	3.6	L HS	L	ATL	Class II

Classification of outcome based on [39]. Abbreviations: CD, cortical dysplasia; Op, operculum; F, frontal; P, parietal; T, temporal; OFC, orbitofrontal cortex; R, right; L, left; ATL, anterior temporal lobectomy; B, bilateral; CD, cortical dysplasia; HA, hippocampal atrophy; HS, hippocampal sclerosis; HH, hippocampal hypertrophy; HI, hippocampal T2/FLAIR intense signal; SAH, selective amygdalo-hippocampectomy; R, right; L, left.

**Table 2:** Autobiographical interview scoring procedure.

Internal Details	Details That Pertain to the Main Event:
	
*Event details*	Happenings: “reactions/emotions in others, the weather, one’s clothing, physical occurrences and actions of others”
	
*Emotion/thought*	“feeling states, thoughts, opinions, expectations, or beliefs” at the time of the event
	
*Perceptual*	“auditory, olfactory, tactile/pain, taste, visual (object details, colours), spatial-temporal (allocentric-egocentric space, body position and duration”
	
*Time*	“Life epoch, year, season, month, date, day of week, time of day, or clock time”
	
*Place*	“countries, bodies of water, provinces, cities, streets, buildings, rooms, and locations within a room”
	
**External Details**	**Details That Do Not Pertain to Main Event:**
	
	Event details, emotion/thought, perceptual, time, place, semantic details, repetitions, other details (metacognitive statements)
	
**Internal-to-total ratio**	**Number of internal details / total number of details**

**Table 3:** Ratings by independent scorers.

Time integration scale	**1 point**—“One or more events that occurred before/after the episode is described, but is limited in terms of specific contextual detail and is lacking global integration” **2 points**—“One or more events that occurred before/after the episode is richly described with specific contextual information but there is no link to a more global time frame” **3 points**—“Episode must be linked to a larger time frame by describing some specific contextual information about at least one event that occurred before or after the recalled event”
AMI rating	**0 points**—“A response based on general knowledge” **1 point**—“A vague personal memory; or an incident that occurred on multiple occasions but no single instance is recalled” **2 points**—“A specific personal memory with few or no details; or a less specific event in which time and place are recalled” **3 points**—“A detailed personal memory that is specific in time and place”
Episodic richness scale	**0 points**- “No episodic information” **1–2 points**—“Limited detail and/or limited elaboration of events” **3–4 points**—“Response has moderate detail and contains at least 2 elaborations” **5–6 points**—“Response is rich in detail, containing at least 2 elaborations, and evokes an impression of true re-experiencing”

**Table 4:** Description of the study sample.

Variable	IR Patients (*n* = 10)		TL Patients (*n* = 15)		Healthy Participants (*n* = 15)		*p* Value	Group Comparison
	Mean (SD)	Min-Max	Mean (SD)	Min–Max	Mean (SD)	Min–Max	(η^2^ or ϕ)	
*Demographics*								
Age (years)	40.3 (6.8)	29–50	34.6 (7.4)	23–49	35.1 (7.1)	26-49	0.126 (0.106)	-
Education (years)	12.9 (2.5)	8–16	12.1 (3.2)	8–17	13.3 (1.8)	11-16	0.403 (0.048)	-
Sex (M/F)	2/8		6/9		6/9		0.517 (0.182)	-
								
*Control variables*								
Similarities Scaled score ^a^	9.8 (3.5)	4–15	9.6 (3.0)	6–14	9.9 (1.4)	7-12	0.942 (0.003)	-
Matrix reasoning Scaled score ^b^	11.4 (3.2)	6–15	11,0 (2.5)	6–15	11.0 (2.4)	6-14	0.918 (0.005)	-
Coding Scaled score ^c^	9.8 (2.4)	6–13	9.2 (3.2)	4–15	9.9 (1.8)	7-13	0.746 (0.016)	-
Memory (story) Raw score ^d^								
Immediate recall	11.5 (6.9)	3–22	10.2 (4.8)	3–19	17.6 (3.9)	11–23	**0.001 (0.318)**	HC > IR = TL
Delayed recall	13.9 (5.1)	8–22	9.8 (4.7)	4–21	19.0 (3.3)	11-22	**<0.001(0.483)**	HC > IR = TL
Emotion recognition Raw score ^e^								
Percent correct (%)	80.3 (7.3)	63–90	76.2 (8.6)	62–90	81.7 (8.2)	60-92	0.182 (0.088)	-
Mean RT (ms)	2836 (748)	1894–4324	3073 (1178)	1342–5396	2517 (899)	1415-4843	0.312 (0.061)	-
BDI-II Raw score	8.4 (6.6)	0–21	12.9 (9.9)	0–30	4.9 (4.1)	0-12	0.017 (0.197)	HC = IR; HC > TL
								
*Epilepsy related factors*								
Age at surgery (years)	34.7 (7.2)	23–49	30.4 (7.8)	19–44	n/a	n/a	0.181 (0.077)	-
Time since surgery (years)	5.6 (2.5)	1.0–9.7	4.2 (2.2)	0.6-8.9	n/a	n/a	0.152 (0.087)	-
Developmental onset (<18 y/o)	7 (70%)		9 (60%)		n/a	n/a	0.610 (0.102)	-

M: male, F: female, ^a–c^ Subtests from the Wechsler Adult Intelligence Scales—4th Edition. ^d^ Based on Sullivan’s short memory test. ^e^ Emotion recognition task. n/a: not applicable.

**Table 5:** Examples of neutral memories evoked by group.

Healthy control	IR patients	TL patients
*party during high school*	*buy a TV*	*buy a new car*
*volunteering day*	*take a driving lesson*	*baptism of daughter*
*car battery failure*	*get the dog to the vet for check up*	*first day at first job*
*help for terrace construction*	*go to a restaurant*	*get hired*
*get the cat to the vet*	*helping someone find a new car*	*have a complete makeover*
*presenting a new boyfriend*	*play in a jam session*	*moving alone for the first time*
*moving to next door flat*	*getting a ticket*	*car accident*
*take an intercity bus*	*go to water slides*	*go to the doctor for annual checkup*
*end of trial period*	*house party*	*adopt a kitten*
*get a compliment*	*make children’s show decorations*	*do an activity with children*
*clean the closet*	*take the train*	*moving out*
*help a friend to get furniture*	*go to the groomer*	*take drugs for the first time*
*old friend become a co-worker*	*go to a conference*	*gardening*
*go to charity show*	*go to a show*	*severe incident with a rented car*
*cooking a new meal*	*get a student job*	*paint walls*
*clean the car*	*go to the doctor for annual checkup*	*meet a breeder’s rare cat*
*miss the bus stop*	*buy chocolate eggs for colleagues*	*get emotional support from friends*
*get to school on a cold day*	*hitchhike*	*get kicked out of family home*
*play role game*	*cook for a dinner with friends*	*meet an individual trainer*
*renew annual transportation card*	*buy a home stereo*	*go to a wedding*
*go shopping*	*do a service exchange*	*presenting new boyfriend to family*
*meeting a professor in the street*	*family dinner*	*first crossfit class*
*etc.*	*etc.*	*etc.*

**Table 6:** AI results after specific probe.

	HC Mean (SD)	IR Patients Mean (SD)	TL Patients Mean (SD)	IR vs. HC *p*-Value (Cohen’s d)	TL vs. HC *p*-Value (Cohen’s d)	IR vs. TL *p*-Value (Cohen’s d)
AI scores						
						
*Internal details*						
Positive	52.45 (22.45)	42.60 (17.24)	30.78 (13.85)	0.409 (0.48)	**<0.001** (1.16)	**0.044** (0.77)
Negative	56.23 (18.58)	42.05 (21.23)	28.74 (13.23)	**0.049** (0.72)	**<0.001** (1.70)	**0.010** (0.79)
Neutral	34.40 (18.82)	34.35 (13.97)	24.44 (10.58)	1.000 (0)	**0.038** (0.65)	**0.021** (0.82)
						
*Internal-to-total ratio*						
Positive	0.78 (0.12)	0.72 (0.15)	0.61 (0.18)	0.822 (0.45)	**0.002** (1.11)	0.080 (0.65)
Negative	0.79 (0.13)	0.72 (0.13)	0.57 (0.20)	0.444 (0.54)	**<0.001** (1.30)	**0.005** (0.85)
Neutral	0.73 (0.15)	0.73 (0.15)	0.63 (0.17)	n/a	n/a	n/a
						
*External details*	15.85 (12.53)	16.22 (12.16)	19.40 (13.93)	n/a	n/a	n/a
						
Internal specific category						
						
*Event details*						
Positive	30.03 (15.89)	26.40 (13.41)	16.68 (10.04)	0.990 (0.24)	**<0.001** (1.00)	**0.009** (0.85)
Negative	34.77 (14.42)	26.05 (16.85)	16.67 (9.78)	0.074 (0.57)	**<0.001** (1.47)	**0.018** (0.72)
Neutral	20.78 (14.39)	20.90 (10.07)	13.91 (6.94)	1.000 (0.01)	**0.049** (0.61)	**0.020** (0.84)
						
*Emotion/thought details*						
Positive	8.28 (4.89)	5.98 (4.13)	5.07 (3.24)	0.271 (0.50)	**0.027** (0.77)	1.000 (0.25)
Negative	8.40 (4.31)	5.65 (3.96)	4.61 (3.71)	0.086 (0.66)	**0.001** (0.94)	0.361 (0.27)
Neutral	3.75 (2.69)	4.13 (2.78)	3.29 (3.25)	n/a	n/a	n/a
						
*Perceptual details*	5.22 (4.65)	3.48 (3.35)	2.67 (2.22)	0.151 (0.42)	**0.006** (0.70)	0.720 (0.30)
						
*Time details*	4.67 (1.77)	4.13 (1.70)	3.21 (1.62)	0.298 (0.31)	**<0.001** (0.86)	**0.016** (0.56)
						
*Place details*	2.47 (1.75)	2.36 (1.60)	2.06 (1.42)	n/a	n/a	n/a
						
Examiner ratings						
						
*Time integration scale*	2.74 (0.50)	2.58 (0.53)	2.22 (0.68)	0.360 (0.31)	**<0.001** (0.87)	**0.005** (0.58)
						
*Episodic richness scale*						
Positive	5.43 (0.81)	4.53 (1.38)	4.03 (1.34)	**0.014** (0.84)	**<0.001** (1.26)	0.438 (0.37)
Negative	5.68 (0.62)	4.43 (1.24)	3.96 (1.30)	**<0.001** (1.37)	**<0.001** (1.69)	0.398 (0.37)
Neutral	4.78 (1.11)	4.30 (1.18)	3.96 (1.40)	0.422 (0.42)	**0.024** (0.65)	0.814 (0.26)
						
*AMI scale*	2.89 (0.31)	2.72 (0.51)	2.52 (0.59)	0.096 (0.42)	**<0.001** (0.79)	**0.042** (0.36)
						

AI scores: mean number of details recalled after specific probe. Examiner ratings: independent scorer ratings after specific probe. n/a: no significant group main or simple effect.

**Table 7:** Subjective self-reported scale.

	HC Mean (SD)	IR Patients Mean (SD)	TL Patients Mean (SD)	Group Simple Effect	IR vs. HC *p* Value (Cohen’s d)	TL vs. HC *p* Value (Cohen’s d)	IR vs. TL *p* Value (Cohen’s d)
**Self-reported scales**							
							
**Emotional intensity**							
Positive	4.82 (1.10)	5.05 (1.01)	5.03 (1.28)	F[2,91] = 0.25, *p* = 0.782	n/a	n/a	n/a
Negative	4.67 (1.32)	4.40 (1.48)	4.37 (1.62)	F[2,93] = 0.49, *p* = 0.615	n/a	n/a	n/a
Neutral	1.13 (1.16)	2.63 (1.84)	2.22 (1.94)	F[2,93] = 9.41, ***p* < 0.001**	**<0.001** (1.02)	**0.008** (0.68)	0.793 (0.22)
							
**Importance at the time**							
Positive	5.32 (1.00)	5.18 (1.15)	5.36 (0.94)	F[2,74] = 0.14, *p* = 0.872	n/a	n/a	n/a
Negative	5.28 (1.18)	5.03 (1.39)	5.09 (1.25)	F[2,76] = 0.33, *p* = 0.723	n/a	n/a	n/a
Neutral	2.10 (1.00)	3.55 (1.55)	3.24 (1.80)	F[2,76] = 10.45, ***p* < 0.001**	**<0.001** (1,17)	**0.003** (0.78)	1.000 (0.18)
							
**Emotional change at the time**							
Positive	4.47 (1.39)	4.53 (1.45)	4.63 (1.10)	F[2,87] = 0.03, *p* = 0.972	n/a	n/a	n/a
Negative	4.95 (1.31)	4.73 (1.20)	4.65 (1.44)	F[2,90] = 0.68, *p* = 0.511	n/a	n/a	n/a
Neutral	1.83 (1.05)	2.90 (1.66)	2.80 (1.66)	F[2,89] = 5.31, ***p* = 0.007**	**0.011** (0.81)	**0.038** (0.70)	1.000 (0.06)
							
				Group main effect			
							
**Importance today**	3.04 (1.95)	3.27 (1.85)	3.39 (1.87)	F[2,40] = 0.45, *p* = 0.642	n/a	n/a	n/a
							
**Rehearsal**	2.78 (1.84)	3.09 (1.94)	3.13 (2.00)	F[2,40] = 0.44, *p* = 0.645	n/a	n/a	n/a
							
**Visualization**	4.48 (1.37)	4.46 (1.35)	4.56 (1.50)	F[2,40] = 0.29, *p* = 0.749	n/a	n/a	n/a
							

n/a: not applicable.

## Data Availability

The data presented in this study are available on request from the corresponding author. The data are not publicly available due to privacy restrictions.
